# Evaluation of Association of Oral Bacterial Profile with HBV and HCV Infection and T Lymphocyte Level in HIV-Positive Patients

**DOI:** 10.1155/2022/8622181

**Published:** 2022-06-23

**Authors:** Fatemeh Lavaee, Farzan Modarresi, Samira Amookhteh, Mohammad Amin Amiri

**Affiliations:** ^**1**^ Oral and Dental Disease Research Center, Oral and Maxillofacial Medicine Department, School of Dentistry, Shiraz University of Medical Sciences, Shiraz, Iran; ^**2**^ Department of Microbiology, School of Medicine, Jahrom University of Medical Sciences, Jahrom, Iran; ^**3**^ Student Research Committee, Shiraz University of Medical Sciences, Shiraz, Iran

## Abstract

**Background:**

This study was aimed to determine the oral bacterial profile of HIV-positive patients and their correlation with T lymphocyte and CD4 count and hepatitis B and C incidence.

**Methods:**

In this study, 73 patients who were diagnosed HIV-positive and were referred to Shiraz HIV research center for routine dental treatment were enrolled. Demographic data including sex, ethnicity, CD4+ T cell, and T lymphocyte counts were collected from their medical records. Supragingival dental plaque and samples from the dorsal of the tongue were collected by sterile swabs. These samples were transferred to the microbiology laboratory of Jahrom University of Medical Sciences. After primary biochemical test of cultured samples, assessment of bacterial biofilms was done by DNA extraction. Real-time PCR with specific primer of each bacterial species was done, and assessment of the results of real time PCR led to determination of the species of the evaluated bacteria. The correlation of bacterial prevalence with hepatitis B and C was evaluated by chi-square test. Furthermore, Mann–Whitney test was used to evaluate the association of bacterial species prevalence with CD4 and T lymphocyte level.

**Results:**

The prevalence of none of the detected bacteria had statistically significant relationship with hepatitis C, except for *Peptostreptococcaceae* (*p* value = 0.016) in the tongue plaque and *Leptotrichia* (*p* value = 0.022) in dental plaque. None of the evaluated bacteria showed any significant association with CD4 and T lymphocytes level, except for *Kingella* (*p* value = 0.025, 0.019, respectively), and also no significant correlation was reported with CD4, except for *Gemella* (*p* value = 0.021) and *Campylobacter gracilis* (*p* value = 0.029).

**Conclusions:**

The diversity of the detected bacteria was more in dental plaque, while their density was more noticeable in the tongue plaque. No significant correlation was found between the prevalence of most of the detected bacteria and CD4 level and T lymphocyte level and incidence of hepatitis B and C.

## 1. Introduction

Human immunodeficiency virus (HIV) infection is a condition associated with progressive immunological response failure, which activates opportunistic life-threatening infections and cancers [1]. Several immunologic factors are involved in this process [2]. In this regard, T cells, especially CD4 cells, can be used as major predictive factors for disease progression during HIV infection [3]. As a result, better understanding of the effects of T cells and CD4 changes and correlative factors are critical in determining the prognosis and treatment planning.

Several studies have demonstrated that the progression of HIV infection can lead to oral pathologies [4–6]. In this regard, CD4 cells seem to play an important role as their reduction beneath 200/*μ*l results in a higher incidence of oral complications [5]. Furthermore, some oral pathologies, which are secondary to significant CD4+ *T* cell reduction, can be the first clinical manifestation of HIV infection [7]. HIV-related immunodeficiency provides proper condition for opportunistic oral pathogens [8]. Moreover, since periodontal diseases are more prevalent among HIV-infected patients, many studies have evaluated the subgingival bacterial profile; however, there are fewer studies assessing the bacterial population patterns in other sites.

In order to evaluate the effect of HIV infection on oral microbial status of the patients, Ferreira et al. [9] have reported greater diversity of subgingival bacteria in HIV-infected patients with chronic periodontal disease. Another study on HIV-positive patients with necrotizing periodontal lesions presented high prevalence of common periodontal pathogens in addition to uncommon species [10]. Furthermore, evaluation of some common periodontal pathogens reported higher prevalence of these pathogens in HIV-seronegative patients than seropositive ones [11]. In addition, the lingual microbiome analysis of untreated HIV-infected patients has revealed an increase in the load of pathogenic bacteria, accompanied by a reduction in commensal species [12]. On the other hand, Kristler et al. [13] revealed the overall similarity of oral microbial profile of HIV-positive and negative patients; although there were some significant differences in the salivary microbiota composition of these two groups. Since the changes in the quantity of bacterial species can result in dental [14] and periodontal pathologies [15], it is important to monitor these changes, especially in immunocompromised patients including HIV-positive patients who need special oral hygiene monitoring [16]. Therefore, the aim of this study is to evaluate the correlation of the oral bacterial profile of HIV-positive patients with the T lymphocyte serum level and the incidence of hepatitis B and C.

## 2. Methods and Materials

### 2.1. Statement of Ethics

This study has been performed according to the principles of Helsinki (Lewis, Jonsson, Kreutz, Sampaio, and van Zwieten-Boot, 2002). The study has been approved by the ethics committee of Shiraz University of Medical Sciences (IR.SUMS.REC.1396.S727).

### 2.2. Subject Selection

In this study, 73 patients who were HIV-positive and referred to Shiraz HIV research center for routine dental treatment during 2018 were enrolled. The subjects who were pregnant, diabetic, or having periodontal diseases; the patients who had used antibiotics or anti-inflammatory medications during the last three months; or the patients who needed antibiotic prophylaxis for dental treatments were excluded.

Inclusion criteria are as follows:

- HIV-positive patients referred for routine dental treatment with no other influencing factor, including other systemic or dental diseases, or antibiotic and anti-inflammatory medications consumption

Exclusion Criteria are as follows:

- The HIV-positive patients who had any other systemic or dental complications, such as diabetes, pregnancy, or periodontal diseases
- Consumption of any antibiotic or other medications that can influence the bacterial population in the oral cavity

Demographic and any related medical data including sex, ethnicity, CD4+ T cell, and T lymphocyte counts, other systemic diseases and type and date of starting antiviral medication were collected from their medical records. Written consent form was taken before the initiation of the study.

### 2.3. Sample Preparation

The supragingival dental plaque and samples from dorsal of the tongue were collected by sterile swabs. These swabs were transferred to the sterile tubes. Sterile swabs were located in a sterile tube or in the thioglycollate medium. These samples were transferred to the microbiology laboratory of Jahrom University of Medical Sciences. The samples were stored in −80 degrees.

After the biochemical test of the primary cultured samples, assessment of bacterial biofilms was done by DNA extraction. Then, in order to detect the bacterial species, real-time PCR with specific primer of each bacterial species was performed. All bacterial species extracted from the collected bacterial biofilm of HIV-positive participants were analyzed and descriptive reports of these participants were prepared.

The swab was then cultured in the culture media of nutrient, blood agar, EMB, Sabouraud agar, MacConkey and CHROM agar, bile-esculin agar, and Mitis Salivarius agar. The swab contained in the tube of aerobic bacteria medium was placed next to three plates contained in the tube of nutrient agar, blood agar, EMB, and MacConkey medium, and the culture was performed once using loop by the streak method. The plates were placed in an incubator of 37°C for 24 to 48 hours. After the incubation period, the bacteria were determined as cocci or bacilli by several factors, including the morphology of the colonies on culture media, presence of hemolysis on blood agar medium, mobility or immobility on the nutrient agar and blood agar medium, the ability to growth on gram-negative bacteria medium, and the smear of bacteria. However, acid-fast bacilli should be incubated for three to four weeks. Biochemical oxidase/catalase tests were done.

The sticky, dark, and smooth colonies of *Streptococcus* mutans were observed in the Mitis Salivarius medium after 48 hours. *Enterococcus faecalis* colonies in Bile-esculin agar medium caused a brown color change in the medium due to the hydrolysis of esculin.

To detect and isolate all types of *Streptococci* from biochemical oxidase tests, growth in 4% and 6.5% NaCl media, acetoin production (VP), esculin hydrolysis, and hemolysis on the blood agar medium were used. In addition, to evaluate the carbohydrate metabolism, we used the phenol red base medium and the sugar. To detect *Lactobacillus* and evaluate the metabolism of carbohydrates, we added sugar to the MRS agar medium without meat and glucose, and bacterial culture was performed by adding phenol red. SIM and egg yolk and gelatin media were used for lecithinase, mobility, and gelatinase tests.

Anaerobic bacteria were also studied along with the detection of aerobic bacteria. Swab tubes and fluid thioglycollate medium were placed for 24 hours at an incubation of 37°C to enrich the anaerobic bacteria present in the sample. They were cultured on nutrient agar, blood agar, EMB, and MacConkey plates using streak method, as in the previous method. Then, all four plates were placed along with the gas pack in the anaerobic jar. After being placed for 48 hours in incubation of 37°C, the plates were studied. In the case of anaerobic bacteria, bacterial morphology was detected by differential tests.

## 3. PCR and Multiplex PCR Technique

### 3.1. Genomic DNA Extraction

In general, the extraction of sufficient and high-quality genomic DNA is one of the most important and precise steps in the molecular works. In this study, DNA extraction kit (Fermentas, Lithuania) was used, and the protocol was performed according to the manufacturer protocol.

### 3.2. Quantitative and Qualitative Study of the Extracted DNA

Two methods can be used to quantitatively and qualitatively study the genomic DNA extracted. First, the quantity of the genomic DNA extracted was measured by a spectrophotometer equipped with ultraviolet light wavelength; then, the agarose gel electrophoresis of the extracted samples was conducted.

### 3.3. Electrophoresis and DNA Detection in the Gel

**Figure 1:**
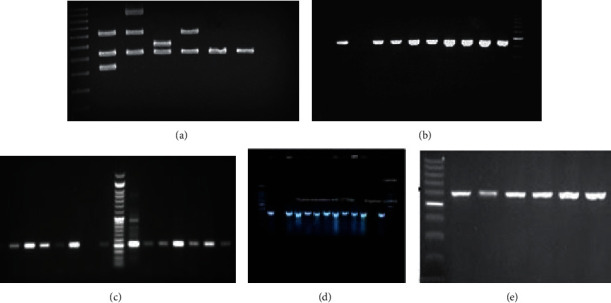
(a): Multiplex PCR amplification of patient isolated bacterial species. (b–e) PCR amplification of patients' isolated: (b) *Streptococcus mutans*, (c) *S. salivarius*, (d) *S. sanguinis*, and (e) *Lactobacillus* species.

The electrophoresis figure and multiplex PCR amplification of patients' isolated bacterial species are presented in Figure 1.

### 3.4. Real-Time PCR Technique

**Figure 2:**
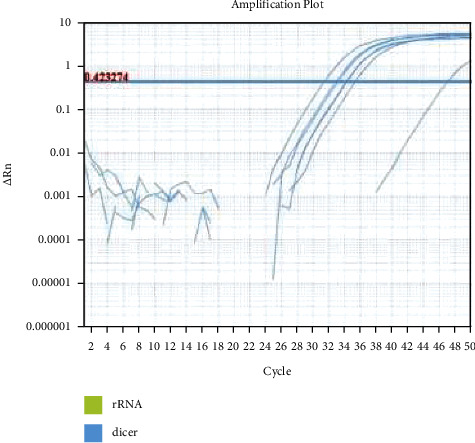
Amplification plot of real time PCR experiment.

RNA extraction was done by using the Cinna Pure RNA Purification Kit. Primers and probes for real-time PCR are represented in Table 1.  Figure 2 shows the amplification plot of real-time PCR experiment.

The correlation of bacterial prevalence with hepatitis C was evaluated by chi-square test, and Mann–Whitney test was used to evaluate the association of bacterial prevalence with CD4 and T lymphocytes level.

## 4. Results

In this study, 73 participants were enrolled; 53.42% of them were men and 46.57% were women. The demographic data are presented in Table 2. The participants were affected by several systemic diseases, in which the most prevalent ones were hepatitis B and C (5.5% and 37.0%, respectively).

The prevalence of the detected bacteria in patients with and without hepatitis C is compared in Table 3. The prevalence of none of the detected bacteria had any statistically significant relationship with hepatitis C, except for *Peptostreptococcaceae* (*p* value = 0.016) in the tongue plaque and *Leptotrichia* (*p* value = 0.022) in the dental plaque. The prevalence of the detected bacteria in the dental plaque and tongue plaque are reported in Table 4.

The presence of the detected bacteria in the evaluated plaque sample was assessed in relation to the serum level of CD4 and T lymphocytes of the participants. All the evaluated bacteria showed no significant relationship with T lymphocyte level, except for *Kingella* (*p* value = 0.019); also, no significant relationship with CD4 was reported, except for *Kingella (pvalue* = *0.025), Gemella* (*p* value = 0.021), and *Campylobacter gracilis* (*p* value = 0.029). The descriptive data of bacterial proportion in dental plaque/tongue plaque is described in Table 5. All evaluated bacteria were more in the tongue plaque than dental plaque, while the diversity of bacteria was more in the dental plaque.

## 5. Discussion

In this study, the bacterial profile of patients' dental and tongue plaque who were HIV-positive was determined by real-time PCR. The diversity of these bacteria was more in the dental plaque than the tongue plaque, while the bacterial number was more in the tongue plaque. Except for some limited detected bacteria, there was no relationship between the prevalence of bacteria and hepatitis B and C, CD4, and T lymphocyte level. The immune-compromised patients are more prone to developing rare microbial infections. The higher prevalence of unusual microbial flora can occur because of the weak immunity of these patients; therefore, investigating the correlation of bacterial profile with the level of immune cells and viral infections in immunocompromised patients such as HIV-positive patients seems necessary [1, 17].

Most of the previous studies on HIV-positive patients were focused on assessing the periodontal pathogenic bacteria. In a study, the oral microbiome in HIV-positive and negative participants in different severities of periodontal disease was reported as follows: *Abiotrophia, Neisseria genus, Kingella,* and *unclassified neisseriaceae* [18].

Contrary to the results of Noguera-Julian et al.'s study on the oral microbiome of HIV-positive patients [18], in the present study, the supragingival plaque was evaluated, in which *Streptococcus* genus*, S. mutans, S. salivarius, S. sanguinis, Lactobacillus, E. faecalis, and Veillonella* were the most prevalent dental and tongue plaque microbiota. Also, some other bacterial species have been detected to be more dominant in the dental plaque or tongue plaque.

As mentioned earlier, the CD4 and T lymphocyte serum levels were not associated with the majority of detected bacteria, except for *Kingella, Gemella, and Campylobacter gracilis*. On the other hand, Lewy et al. [19] have reported a positive correlation between CD4 cells and *Streptococcus* and *Lactobacillus.* This difference can be attributed to the differences in study design and the sampling method. These findings are not completely confirmed by previous studies, in which no association was reported for all the detected bacteria [18, 20].

As reported in other studies, differences in the structural composition are associated with anatomic sampling sites [13, 18], which was confirmed in this study. In this study, we found that the bacterial density in the tongue plaque was more than that in the dental plaque, while the variety of these detected bacteria was more in the dental plaque. The tongue plaque was depleted of some bacteria such as *Actinobacterium, Synergistates, Eubacterium, Abiotrophia, Parumonas,* and *Leptotrichia.* Additionally, some others were significantly dominant in the dental plaque in comparison with the tongue plaque. In spite of the effect of the location on bacterial composition of oral flora, Scully et al. [20] reported that the depth of pockets did not have a significant impact on the type of bacterial species during the sampling process.

Also, various bacterial profiles have been reported in different studies [18, 21]. In several studies, different subgingival bacterial profiles in HIV-positive patients with periodontal diseases were reported. The most common ones are *Prevotella nigrescens, Campylobacter rectus, P. nigrescens, P. gingivalis,* and *T. denticula* [22]. However, in a study by Aas et al. [23], *Gemella, Dialister, Streptococcus,* and *Veillonella* were reported as the most prevalent species in HIV-positive patients with periodontal disease. Furthermore, in a study by Scully et al. [20], the bacterial profile of HIV-seropositive patients mainly was *Actinobacillus actinomycetemcomitans (A.a)*, *Porphyrom*onas *gingivalis, Campylobacter rectus, Provotella intermedia,* and *Fusobacterium nucleatum*.

Aside from the variations reported in the bacterial community of HIV-positive patients, it is also important to note the fluctuations in the bacterial pathogens during the disease progression. In this regard, Lewy et al. [19] have reported that enhanced serum level of HIV RNA is associated with an increasing trend in *Prevotella* and *Veillonella*, accompanied by decline in the *Streptococcus* and *Lactobacillus* count. This indicates a possible risk of developing periodontal diseases; however, Guo et al. [24] have failed to demonstrate a relationship between the periodontal status and different stages of the disease. In addition, they have exhibited a decreasing pattern in the *Porphyromonas* population during the increase in the blood viral load of HIV. These transitions in the oral flora can play a role in the immune regulation of patients with HIV [21]. This is in line with abovementioned association of *Kingella, Gemella,* and *Campylobacter gracilis* with CD4 and T lymphocytes in our study, which highlights the importance of monitoring these changes over time. As a result, targeting the oral microbiome and inflammatory status can be a possible logical approach in controlling the long-term immune imbalance in patients with HIV [21].

The controversies in the abovementioned studies can be related to different methods of plaque sampling from different sites with different periodontal health status. The inflammatory status of the sampling site can affect the bacterial composition. Since most HIV-positive patients receive highly active antiretroviral therapy (HAART), the microbial diversity in the samples of HIV-positive subjects has been reported to be reduced [13].

As mentioned earlier, the fluctuations in the quantity of bacterial species in the oral flora can, in some instances, lead to periodontal [25] and malignant lesions [26]. This effect can be amplified when accompanied by other factors including NLRP3 in developing periodontitis [27] and genetic polymorphisms, such as MTHFR mutations, which is an important factor to consider for oral cancer [28]. Furthermore, the patients' periodontal biotype is of critical importance in the prognosis of periodontal diseases [29, 30]. In general, in order to improve the oral health status of patients with HIV infections, aside from considering only oral bacterial profile fluctuations, we recommend the comprehensive evaluation of the patients' other risk factors.

In this study, due to the funding issues, 30 species of common oral bacteria were evaluated, which can be considered as a limitation. To better confirm the outcomes of this study, larger sample sizes with a wider spectrum of bacterial evaluation is recommended for the future studies.

## 6. Conclusion

The following conclusions were drawn from this study:

1. There was no significant relationship found between the prevalence of most of the detected bacterial species with CD4 level, T lymphocyte level, and hepatitis B and C incidence, except for some limited species in HIV-positive patients.
2. The diversity of the detected bacteria was more in the dental plaque, while their density was more noticeable in the tongue plaque.
3. Some bacteria such as *Streptococcus* genus*, S. mutans, S. salivarius, S. sanguinis, Lactobacillus, E. faecalis,* and *Veillonella* were very prevalent in both tongue and dental plaques.
4. In addition, some other bacterial species such as *Actinobacterium, Synergistates, Eubacterium, Abiotrophia, Parumonas, Leptotrichia, Solobacterium,* and *Dialister* were dominant in the dental plaque, while the tongue plaque was depleted from the mentioned bacteria.

## Figures and Tables

**Table 1:** Primers and probes for real-time PCR.

Name of bacteria	Primers and probes for real-time PCR	Annealing
*S. salivarius*	Forward primer: MKK-GTGTTGCCACATCTTCACTCGCTTCGG Reverse primer: MKK- CGTTGATGTGCTTGAAAGGGCACCATT	54
*S. mutans*	Forward primer: 5-GGCACCACAACATTGGGAAGCTCAGTT Reverse primer: 5-GGAATGGCCGCTAAGTCAACAGGAT	53
*S. sanguinis*	Forward primer: GGATAGTGGCTCAGGGCAGCCAGTT Reverse: GAACAGTTGCTGGACTTGCTTGTC	55
*Lactobacillus*	Forward primer: AGAGTTTGATTGGCTCAG Reverse: CACCGCTACACATGGAG	52
*E. faecalis*	Forward: 5-ATC AAG TAC AGT TAG TCT TTA G-3 Reverse:5-ACG ATT CAA AGC TAA CTG AAT CAG T-3	55
*Actinomyces*	5-AGCGCTTGCCTTTTTGGTG-3 (annealing 53) 5-AACCCGCCATGCGACAGACCCG-3	56
*Fusobacterium periodonticum*	5-ACCTTATCAAGACTTATT ATT TC-3 (annealing 55) 5-TCA AAC TCT ATY TCA GGA ACA A-3	52
*Bacteroides*	GTTTAATTCGATGATACGCGAG TTAASCCGACACCTCACGG	52
Actinobacteria	TGTAGCGGTGGAATGCGCAATTAAGCCACATGCTCCGCT	55
*Campylobacter gracilis*	5'-AAC GGA ATT TAA GAG AGC TT-3′ 5′-CTT TCC CGA TTT ATC TTA TG	54
*Genus Campylobacter*	AGTCTTGGCAGTAATGCACCTAACGATATGCCATTGTAGCACGTGTGTCG	52
*Peptostreptococcaceae*	ACTCCTACGGGAGGCAGCAGGACTACTTGGGTATCTAAT	56
*Synergistetes*	GAGTACCGGAGAGGCAAGTGAGTTACCGTCCAGCAAGTCG	56
*Neisseriaceae*	AACATATCGGAACGTACC (probe)	50
*Eubacteria*	AGAGTTTGATCATGGCTCAGGCTGCTGGCACGAAGTT	54
*Staphylococcus aureus*	CATGGTTCAAAAGTGAAAGAC (probe)	52
*Streptococcus genus*	GCGGGGGATAACTATTG (probe)	52
*Corynebacterium genus*	CGAAGCTTTTGTGACGG (probe)	52
*Enterobacter genus*	AGGAAGGTGTTGTGGTTAA (probe)	55
*Abiotrophia*	GCGTGAATGCCATCTATCAGACTTGTTGGTCTCGCAGTCA	57
*Kingella*	TGCCAAAGTAAAACCAGCTGAAAACTTACCTAATTTTGGCAAAGCAA	55
*Prevotella*	CACTGTAAACGATGGATGCCCCAGACGTTGGGCTGGTTTA	58
*Veillonella*	GAAAGAAGCGCGCACCGACAGTGTGTAACAAGGGAGTACGGACC	55
*Rothia*	GGGTTGTAAACCTCTGTTAGCATCCGTACCCACTGCAAAACGCAG	52
*Parvimonas*	AGAGTTTGATCCTGGCTCAGACGGCTACCTTGTTACGACTT	50
*Leptotrichia*	GAGTTTGATYCTGGCTCAGAAGGAGGTGATCCAACCGCA	59
*Solobacterium*	AGTTTGATCCTGGCTCAGCTTGTTACGACTTCACCC	55
*Gemella*	AGAGTTTGATCCTGGCTCAGACGGCTACCTTGTTACGACTT	54
*Haemophilus*	AGAGTTTGATCTGGCTCAGGACGGGCGGTGTGTACA	50
*Dialister*	TTC TAA GCA TCG CAT GGT GCGAT TTC GCT TCT CTT TGT TG	57

**Table 2:** The demographic data of HIV-positive participants.

HIV positive patients	Number	Mean age (years-old)	Mean values of CD4 (per *μ*l)	Mean values of T lymphocyte (per *μ*l)
Men	39 (53.42%)	39.1 ± 10.44	474.48 ± 223.554	2.136 ± 0.71
Women	34 (46.57%)	39.14 ± 8.59		
Total	73 (100%)	39.12 ± 9.56		

**Table 3:** The prevalence of the detected bacteria in patients with and without hepatitis C.

Dental bacteria	Tongue plaque			Dental plaque		
	Hepatitis C positive	Hepatitis C negative	*p* value	Hepatitis C positive	Hepatitis C negative	*p* value
*S. salivarius*	96.3	95.7	0.693	100	95.7	0.394
*S. mutans*	100	97.8	0.630	100	97.8	0.630
*S. sanguinis*	92.6	89.1	0.483	100	93.5	0.244
*Lactobacillus*	85.2	87.0	0.546	85.2	78.3	0.344
*E. faecalis*	81.5	82.6	0.570	74.1	58.7	0.142
*Actinomyces*	22.2	26.1	0.470	59.3	41.3	0.107
*Fusobacterium periodonticum*	29.6	23.9	0.393	51.9	50.0	0.536
*Bacteroides*	3.7	2.2	0.606	37.0	26.1	0.235
*Actinobacteria*	100	100		29.6	43.5	0.178
*Campylobacter gracilis*	3.7	6.5	0.526	33.3	43.5	0.273
*Genus Campylobacter*	7.4	15.2	0.277	59.3	73.9	0.149
*Peptostreptococcaceae*	14.8	0.0	0.016	40.7	41.3	0.580
*Synergistetes*	No	No		18.5	15.2	0.476
*Neisseriacceae*	44.4	26.1	0.089	85.2	80.4	0.430
*Eubacteria*	No	No		25.9	28.3	0.527
*Staphylococcus aureus*	33.3	43.5	0.273	59.3	76.1	0.107
*Streptococcus genus*	100	100		100	100	
*Corynebacterium genus*	18.5	23.9	0.409	48.1	45.7	0.514
*Enterobacter genus*	7.4	13.0	0.372	48.1	52.2	0.464
*Abiotrophia*	No	No		37.0	23.9	0.176
*Kingella*	11.1	4.3	0.261	29.6	32.6	0.502
*Prevotella*	3.7	13.0	0.188	40.7	37.0	0.469
*Veillonella*	74.1	76.1	0.530	92.6	100	0.134
*Rothia*	3.7	2.2	0.606	25.9	32.6	0.372
*Parvimonas*	No	No		25.9	26.1	0.607
*Leptotrichia*	No	No		33.3	10.9	0.022
*Solobacterium*	No	No		11.1	6.5	0.391
*Gemella*	3.7	19.6	0.054	11.1	10.9	0.628
*Haemophilus*	14.8	13.0	0.546	18.5	10.9	0.282
*Dialister*	No	No		3.7	6.5	0.526

**Table 4:** The prevalence of different bacteria in the dental plaque and tongue plaque.

Bacteria name	The prevalence of detected bacteria in tongue plaque (percent)	The prevalence of detected bacteria in dental plaque (percent)
*S. salivarius*	95.9	97.3
*S. mutans*	98.6	98.6
*S. sanguinis*	90.4	95.9
*Lactobacillus*	86.3	80.8
*E. faecalis*	82.2	64.4
*Actinomyces*	24.7	47.9
*Fusobacterium periodonticum*	26.0	50.7
*Bacteroides*	2.7	30.1
*Actinobacteria*	0.0	38.4
*Campylobacter gracilis*	5.5	39.7
*Genus Campylobacter*	12.3	68.5
*Peptostreptococcaceae*	5.5	41.1
*Synergistetes*	0.0	16.4
*Neisseriaceae*	32.9	82.2
*Eubacteria*	0.0	27.4
*Staphylococcus aureus*	39.7	69.9
*Streptococcus genus*	100	100
*Corynebacterium genus*	21.9	46.6
*Enterobacter genus*	11.0	50.7
*Abiotrophia*	0.0	28.8
*Kingella*	6.8	31.5
*Prevotella*	9.6	38.4
*Veillonella*	75.3	97.3
*Rothia*	2.7	30.1
*Parvimonas*	0.0	26.0
*Leptotrichia*	0.0	19.2
*Solobacterium*	0.0	8.2
*Gemella*	13.7	11.0
*Haemophilus*	13.7	13.7
*Dialister*	0.0	5.5

**Table 5:** The descriptive data regarding the proportion of bacteria in dental plaque/tongue plaque.

	N	Minimum	Maximum	Mean	Std. deviation
*S. salivarius*	68	0.03	1.60	0.6732	0.34005
*S. mutans*	71	0.25	2.00	0.8500	0.33722
*S. sanguinis*	63	0.1	2.0	0.913	0.3035
*Lactobacillus*	50	0.25	1.50	0.8240	0.26597
*E. faecalis*	38	0.20	1.50	0.7461	0.32682
*Actinomyces*	7	0.5	1.0	0.829	0.2360
*Fusobacterium periodonticum*	9	0.5	1.0	0.833	0.2121
*Actinobacteria*	0				
*Campylobacter gracilis*	1	1.0	1.0	1.000	
*Genus Campylobacter*	6	0.5	1.0	0.817	0.1835
*Peptostreptococcaceae*	0				
*Synergistetes*	0				
*Neisseriaceae*	21	0.5	2.5	1.000	0.4370
*Eubacteria*	0				
*Staphylococcus aureus*	25	0.25	1.20	0.8440	0.259553
*Streptococcus genus*	73	0.20	4.00	0.8336	0.65765
*Corynebacterium genus*	10	0.2	1.0	0.760	0.2633
*Enterobacter genus*	5	1.0	1.0	1.000	0.0000
*Abiotrophia*	0				
*Kingella*	1	0.5	0.5	0.500	.
*Prevotella*	3	0.8	1.0	0.867	0.1155
*Veillonella*	53	0.25	2.00	0.9085	0.26050
*Rothia*	2	1.0	1.2	1.100	0.1414
*Parvimonas*	0				
*Leptotrichia*	0				
*Solobacterium*	0				
*Gemella*	0				
*Haemophilus*	2	0.8	1.2	1.000	0.2828
*Dialister*	0				
Valid N (listwise)	0				

## Data Availability

The datasets of this study are available by the corresponding author in a reasonable request.

## Abbreviations

HBV: Hepatitis B virus
HCV: Hepatitis C virus.
